# Efgartigimod as a potential alternative to intravenous immunoglobulin in Guillain-Barré syndrome: a retrospective study

**DOI:** 10.3389/fimmu.2026.1734853

**Published:** 2026-04-14

**Authors:** Changyue Liu, Kaiyue Wang, Shimeng Zhang, Naiyong Gao, Guangyin Xu, Faying Qi

**Affiliations:** Department of Neurology, Fourth Ward, Linyi People’s Hospital, Linyi, Shandong, China

**Keywords:** efficacy, Guillain-Barré syndrome, ophthalmoplegia, outcome, respiratory dysfunction

## Abstract

**Purpose:**

A disabling peripheral nervous system disorder, Guillain-Barré syndrome (GBS), has very limited treatment options. However, some patients have poor outcomes even after receiving treatment. This study aimed to compare the efficacy of efgartigimod versus intravenous immunoglobulin (IVIG) for treating GBS with disability.

**Patients and methods:**

We retrospectively analyzed data from GBS patients who were unable to walk independently and were admitted between January 1, 2023, and February 28, 2025. Our primary outcome was the proportion of patients able to walk independently at week 4 and able to run at week 12.

**Results:**

After including 34 patients in the study, we divided them into two groups: 12 in the efgartigimod group and 22 in the IVIG group. Patients on efgartigimod displayed numerically better short-term (100% vs. 64%, *p* = 0.03) and long-term (100% vs. 55%, *p* = 0.006) outcomes than those treated with IVIG.

**Conclusion:**

Efgartigimod showed favorable safety and clinical efficacy for treating GBS with disability in this single-center retrospective study, with notable clinical improvement observed in patients with ophthalmoplegia and respiratory insufficiency. Larger prospective studies are needed to validate these preliminary findings.

## 1 Introduction

A common immune-mediated multiple polyradiculoneuropathy, Guillain-Barré syndrome (GBS), is characterized by symmetrical limb paralysis and decreased or absent deep tendon reflexes, with approximately 100,000 new cases reported per year. Clinical manifestations vary in severity and can be life-threatening in severe cases due to respiratory failure, severe autonomic dysfunction, etc. Therefore, immunotherapy should be initiated immediately in severely ill patients (1, 2).

The current first-line treatment for GBS includes intravenous immunoglobulin (IVIG) and plasma exchange (PE) (3, 4). However, 20% of patients are unable to walk unaided one year after the onset of the disease, and up to 5% die post-treatment (1). Moreover, PE is not recommended for patients with autonomic dysfunction or severe infections, while IVIG is contraindicated in those with acute renal failure. Hence, their availability and demand for usage are limited in specialized facilities.

Antibody-mediated humoral immune processes are crucial for the pathogenesis of GBS. Immunoglobulin G (IgG) maintains its long serum half-life via FcRn-mediated recycling that relies on pH-dependent binding affinity. FcRn binds to the Fc region of endocytosed IgG in the acidic microenvironment of endocytic vesicles (pH 5.0-6.5), thereby rescuing IgG from lysosomal degradation, upon exposure to the neutral extracellular physiological environment (pH 7.4), the binding affinity between FcRn and IgG is markedly reduced, which results in the release of IgG back into the circulation and the completion of the entire recycling process (5). Efgartigimod is a rationally engineered FcRn antagonist with an ABDEG-modified Fc domain that enhances its pH-dependent binding affinity for FcRn. It competitively outcompetes endogenous pathogenic IgG for FcRn binding in acidic endosomes, blocking IgG recycling and diverting unbound pathogenic IgG to lysosomal degradation, which results in a dose-dependent reduction of total serum IgG levels and exerts a targeted therapeutic effect on IgG-mediated autoimmune diseases such as GBS (6). Animal model studies and case reports confirm that efgartigimod is a potential therapeutic treatment for GBS (7–10). However, none of the current studies have evaluated the efficacy of efgartigimod for treating GBS. Therefore, our study aimed to compare the efficacy of efgartigimod and IVIG in GBS patients with disability.

## 2 Materials and methods

### 2.1 Patients and data collection

We retrospectively analyzed the medical records of patients who fulfilled the diagnostic criteria of GBS or one of its variants and were admitted to the Neurological unit of Linyi People’s Hospital, from 1st January 2023 to 28th February 2025.

Our inclusion criteria were a diagnosis of GBS based on the National Institute of Neurological Disorders and Stroke criteria (11), patients aged ≥18 years, individuals within 14 days of onset of illness, and GBS disability score (GBS-DS) ≥3 at the peak of illness. The exclusion criteria included patients with severe neurological dysfunction caused by other diseases, severe infections, or serious concurrent illnesses (such as tumors, severe liver and kidney function impairment) caused by other diseases, as well as those who received treatment with PE and mechanical ventilation. The GBS-DS was defined as follows: 0, healthy; 1, having minor symptoms or signs but fully capable of manual work; 2, able to walk ≥10 m independently; 3, able to walk ≥10 m with a walker or support; 4, bedridden or chairbound; 5, requiring assisted ventilation at least part of the day; and 6, dead (12).

We collected the patients’ baseline clinical data, including demographic (age and sex), anamnestic (date of disease onset, and previous events, such as infections, diarrhea, etc.), clinical features (muscle strength, subjective and objective sensory impairments, autonomic dysfunction, cranial nerve impairment, bulbar palsy and respiratory insufficiency), electrophysiological examination findings, GBS-DS and Medical Research Council (MRC) sum scores. For patients with complete electrophysiological examination findings, they were classified into four electrophysiological subtypes: AMAN, AMSAN, AIDP, and unclassified (1). Nerve conduction studies were performed in accordance with the guidelines of the European Academy of Neurology and Peripheral Nerve Society (13).

### 2.2 Interventions

At admission, patients were fully informed about the efficacy of different treatment options, and the treatment strategy was selected by the patients. IVIG was administered at 0.4 g/kg per day for 5 consecutive days via intravenous infusion; efgartigimod was given at 10 mg/kg per administration, with subsequent doses decided by clinical treatment response, an interval of 3–7 days between infusions, and a maximum of 4 administrations in total.

### 2.3 Outcomes

Our primary outcome was the proportion of patients with GBS-DS ≤ 2 and GBS-DS ≤ 1 at weeks 4 and 12 from symptom onset, respectively. Secondary outcomes included: (1) the time to improvement by at least one functional grade; (2) changes in MRC sum scores from peak to improvement by one functional grade; (3) changes in GBS-DS from peak disability to weeks 4 and 12.

### 2.4 Statistical analysis

Data were statistically analyzed using SPSS 26.0 software, and continuous variables were evaluated by the K-S normality test. Normally distributed data were described as the mean ± standard deviation, and intergroup comparisons were made using the t-test. Non-normal measurements were expressed as medians and quartiles, and were assessed by the Mann-Whitney U test. Categorical variables were described by frequencies and percentages, and intergroup comparisons were conducted using the four-cell chi-square test or Fisher’s exact probability method. The GBS and the GBS-DS scores of ≤2 at 4 treatment weeks and ≤1 at 12 weeks of treatment were used as the dependent variables, respectively. Furthermore, the independent variables with *p* < 0.05 in the univariate results were included in multivariate logistic analyses. Statistically significant differences were indicated by values of *p* < 0.05.

## 3 Results

### 3.1 Patients

After enrolling 34 patients in this study, we divided them into two treatment groups: 22 receiving IVIG and 12 receiving efgartigimod. The mean time from the onset of illness to drug administration was 6·3 days (2–12) and 8 days (3–15) in the efgartigimod and IVIG groups, respectively. Three were diagnosed with MFS-GBS overlap syndrome in the efgartigimod group. Two of these cases had severe limb weakness (MRC sum scores = 36), and one was diagnosed with Bickerstaff’s brainstem encephalitis with severe somnolence on admission. Detailed baseline demographic and clinical characteristics are presented in **Table 1**.

**Table 1:** Patients’ baseline characteristics.

Variables	Efgartigimod (n = 12)	IVIG (n = 22)	*P*
Age	50 (24-77)	57 (24-75)	0.417
Sex			0.005
Men	9 (75)	5 (23)	
Women	3 (25)	17 (77)	
Antecedent illness			
None	5 (42)	5 (23)	0.217
Diarrhea	2 (17)	5 (23)	>0.999
Respiratory tract infection	4 (33)	11 (50)	0.476
Other	1 (8)	3 (14)	>0.999
Days from onset to drug administration	6.33 ± 3.52	8.00 ± 3.27	0.176
MRC sum scores	40.0 (36.0, 50.5)	36.0 (31.3, 40.8)	0.012
GBS-DS			0.514
3	7 (58)	11 (50)	
4	5 (42)	9 (41)	
5	0 (0)	2 (0.9)	
Clinical symptoms			
Muscular hyposthenia	12 (100)	22 (100)	>0.999
Paresthesia	11 (92)	18 (82)	0.635
Cranial nerve involvement	8 (67)	9 (41)	0.282
Autonomic nerve involvement	3 (25)	6 (27)	>0.999
Respiratory insufficiency	2 (17)	5 (23)	>0.999
Clinical subtypes			0.121
Typical GBS	8 (67)	17 (77)	
Pure motor	2 (17)	4 (18)	
MFS	0 (0)	1 (5)	
MFS-GBS overlap syndrome	3 (25)	0 (0)	
Electrophysiological subtypes	n=7	n=20	0.666
AIDP	3 (43)	7 (37)	
AMAN	2 (29)	8 (42)	
AMSAN	2 (29)	2 (11)	
unclassified	0 (0)	2 (11)	
The mean times*	10 (6,11)	9 (7,11.25)	0.759
CSF	n=5	n=7	
WBC	3 (2.25, 3)	3 (1, 3.5)	0.765
Protein (mg/L)	1115 (934.25, 1194.5)	998 (706, 2291.5)	0.836
albumin cytological dissociation	5 (100)	6 (86)	>0.999
The mean times*	10.5 (8.5,11)	9 (8.12)	0.885

Results presented as number (% column), mean ± SD, or median (25–75 percentile range).

GBS-DS, GBS disability score; IVIG, intravenous immunoglobulin; MRC, Medical Research Council; AIDP, acute inflammatory demyelinating polyneuropathy; AMAN, acute motor axonal neuropathy; AMSAN, acute motor-sensory axonal neuropathy; CSF, cerebrospinal fluid; WBC, white blood cells.

*From symptom onset to performing electrophysiological examination and lumbar puncture.

### 3.2 Outcomes

**Figure 1:**
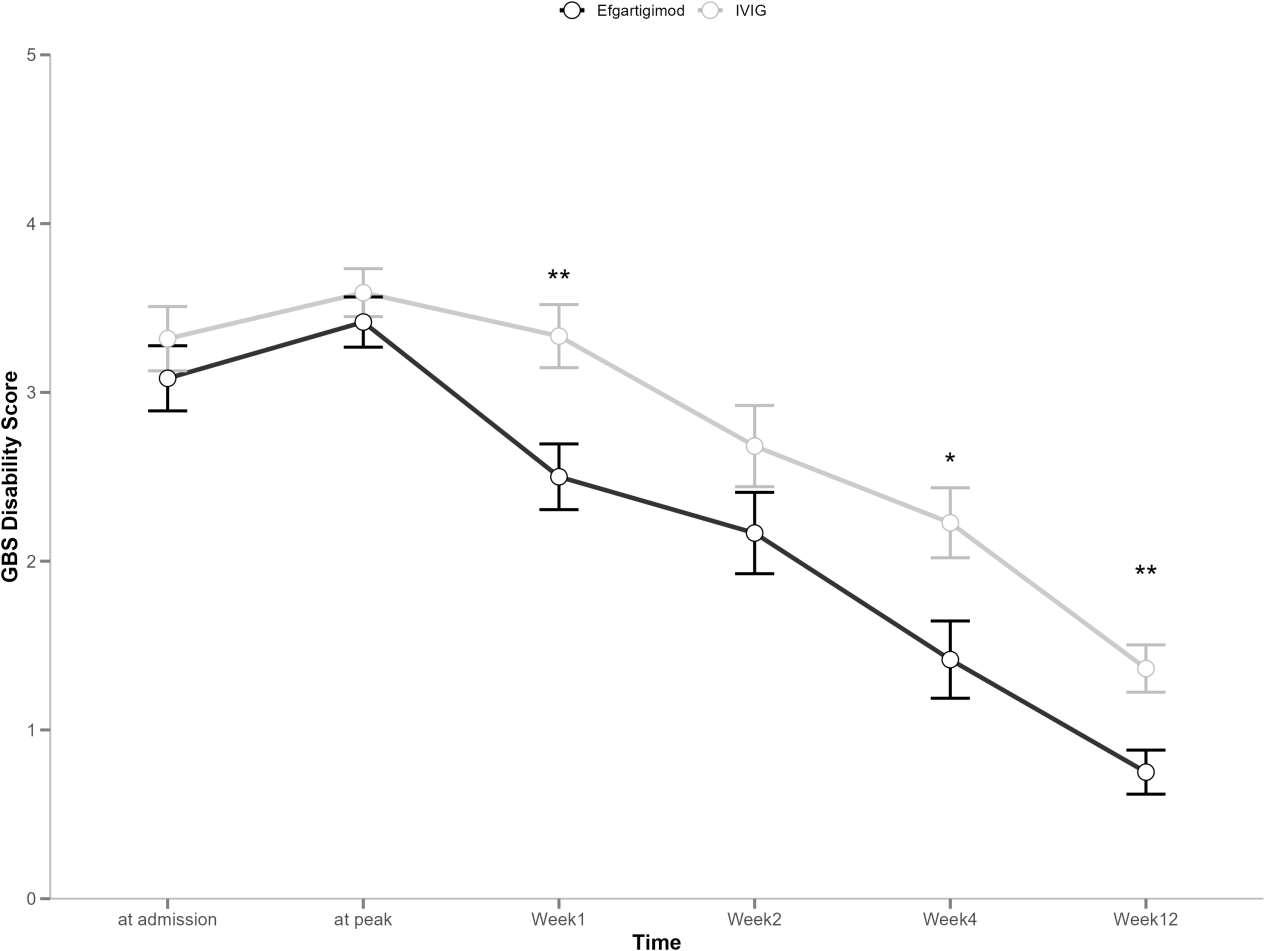
Mean change from the baseline in GBS disability scores. Error bars represent the mean ± standard deviation (SD); Asterisks indicate statistical significance compared to baseline: *P < 0.05, **P < 0.01.

**Figure 2:**
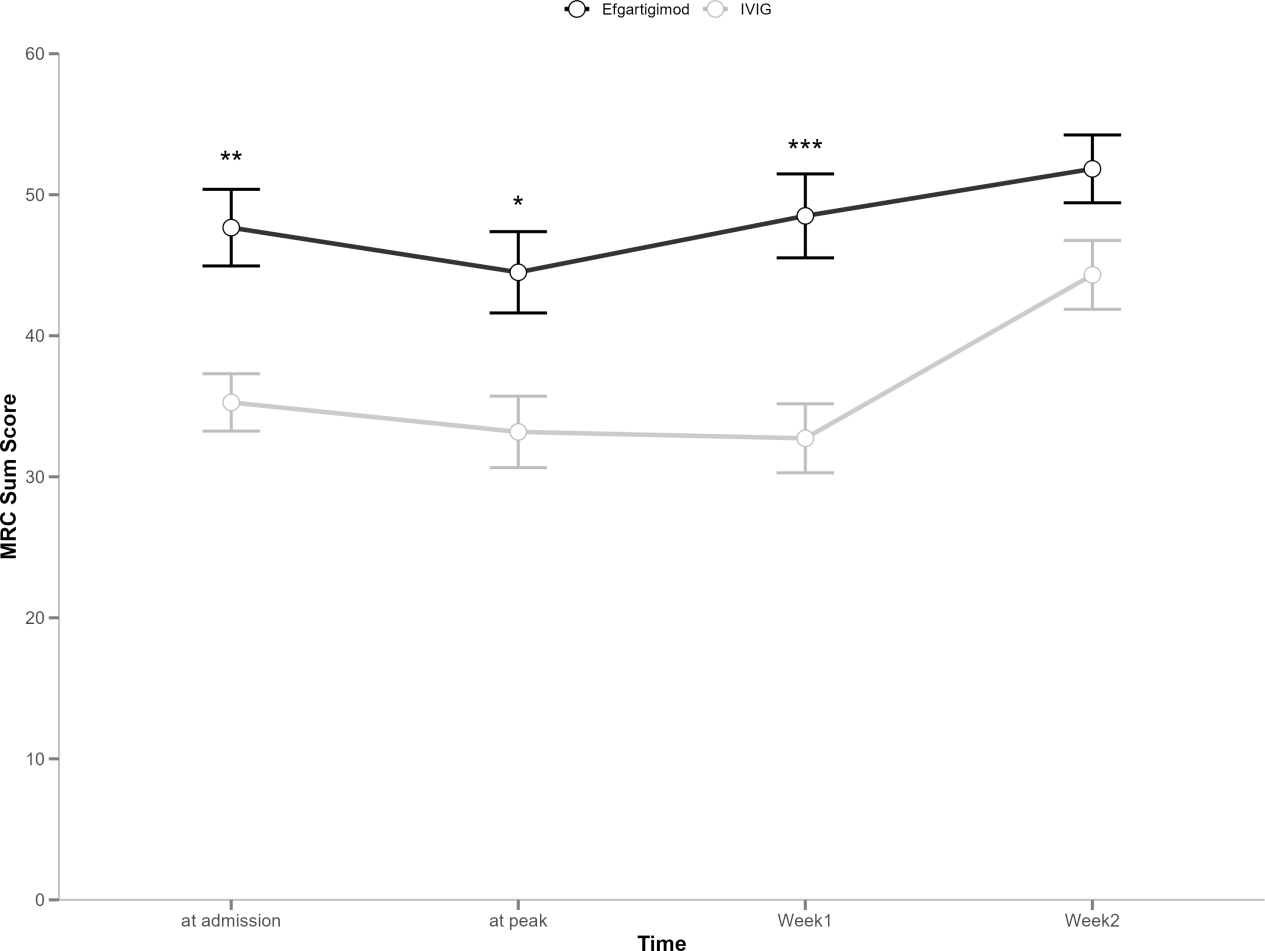
Mean change from baseline in MRC sum scores. MRC, Medical Research Council; Error bars represent the mean ± standard deviation (SD); Asterisks indicate statistical significance compared to baseline: *P < 0.05, **P < 0.01, ***P < 0.001;.

**Figure 3:**
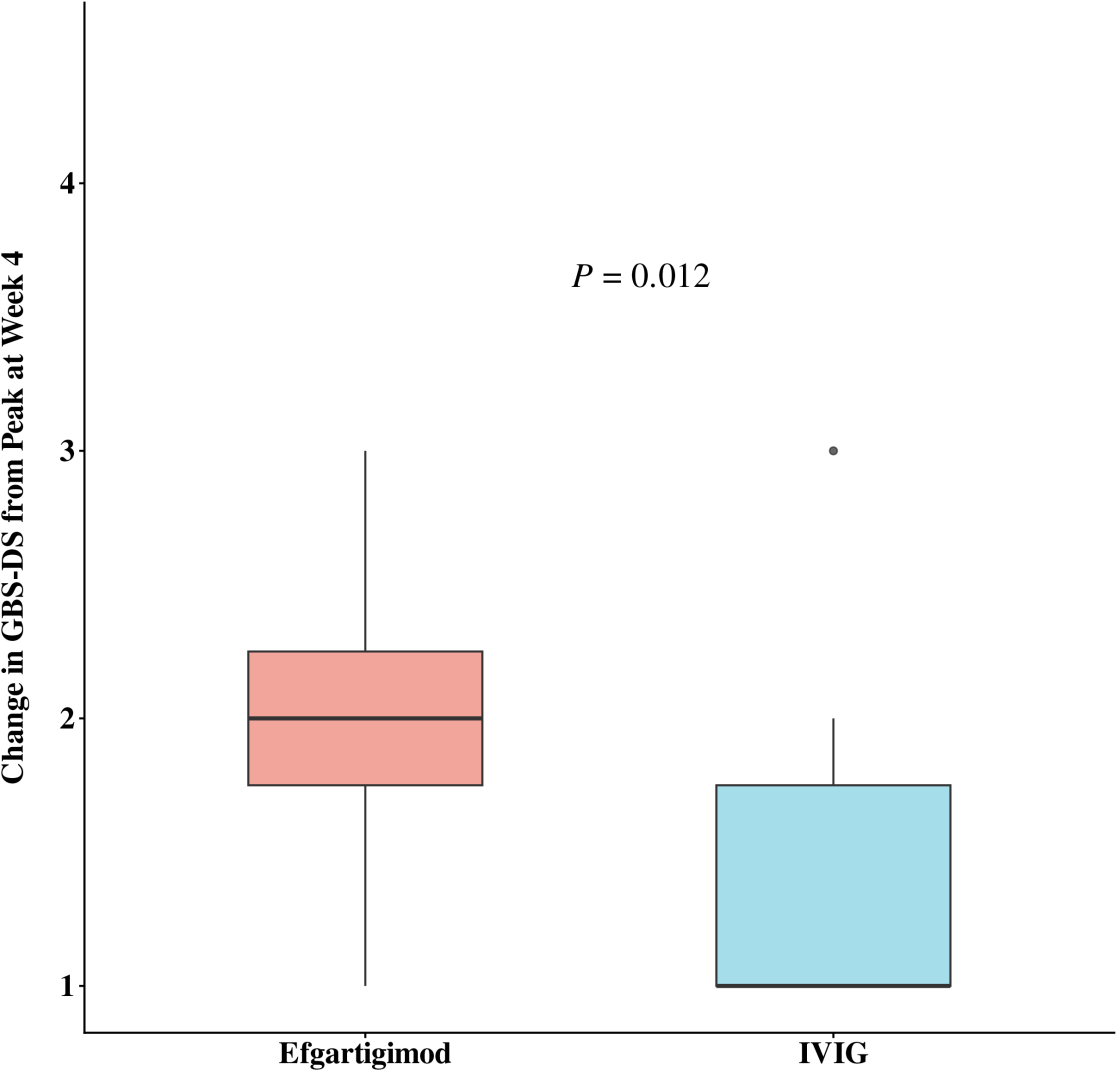
Change in GBS-DS from peak at week 4.

**Figure 4:**
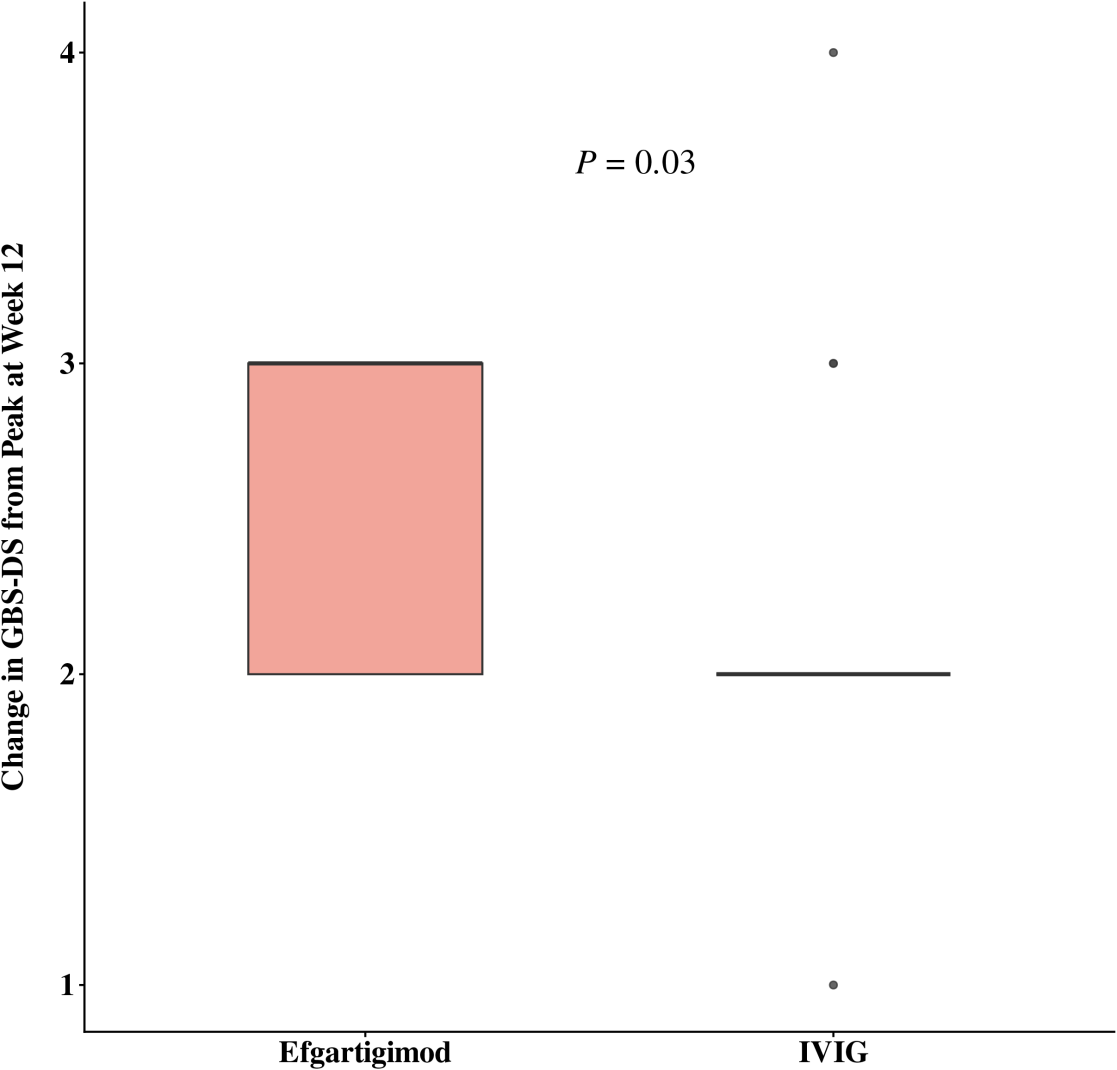
Change in GBS-DS from peak at week 12.

**Table 2** shows the comparative differences in efficacy between efgartigimod and IVIG. The proportion of patients with GBS-DS ≤ 1 at week 12 was 100% (n=12) and 55% (n=12) in the efgartigimod and IVIG groups, respectively, with a significant difference (*p* = 0.006). The number of days required to improve one functional level was numerically less in the efgartigimod group compared to the IVIG group, regardless of whether this improvement was measured from the onset of disease (10 days vs. 13.5 days) or the start of treatment (3.5 days vs. 4.0 days). In terms of improved MRC sum scores, the efgartigimod group (6.0) showed more efficacy than the IVIG group (5.5); however, the above differences were not significant. **Figures 1** and **2** display changes in the mean scores of different clinical rating scales. Moreover, the clinical rating scores of the efgartigimod group were superior to the IVIG group at weeks 1 (**Figures 1** and **2**), 4, and 12 (**Figure 1**), respectively. **Figures 3** and **4** illustrate the changes in GBS-DS from peak disability to week 4 and week12 across treatment groups. **Figure 3** elaborates that the efgartigimod group (2, IQR 1.75–2.25) has a higher median improvement at week 4 compared with the IVIG group (1, IQR 1.00–1.75; *p* = 0.012). A consistent trend is observed at week 12 (3, IQR 2.00–3.00 vs. 2, IQR 2.00–2.00; *p* = 0.030) (**Figure 4**).

**Table 2:** Comparative analysis of outcome indicators between the efgartigimod and IVIG groups.

Outcome Measures	Efgartigimod (n = 12)	IVIG (n = 22)	*P*
Change in MRC sum scores*	6.0 (0.0, 12.0)	5.5 (4.0, 11.3)	0.662
Time to improve by one functional grade (days)			
from the onset of disease	10.0 (7.8, 13.5)	13.5 (10.0 16.8)	0.100
from drug administration	3.5 (2.0, 5.3)	4.0 (3.0, 5.8)	0.583
GBS-DS ≤ 2			
Week 4	12 (100)	14 (64)	**0.030**
Week12	12(100.00)	12 (100.00)	>0.999
GBS-DS ≤1			
Week4	5 (42)	4 (18)	0.224
Week 12	12 (100)	12 (55)	**0.006**

Results presented as number (% column), median (25–75 percentile range).

GBS-DS, GBS disability score; IVIG, intravenous immunoglobulin; MRC, Medical Research Council.

*From peak to improvement by one functional grade. Bold values indicate statistical significance.

The logistic regression results are displayed in **Tables 3** and **4**. Univariate regression analysis showed that the MRC sum scores at admission (OR = 1.13, *p* = 0.03) and peak (OR = 1.16, *p* = 0.016) were associated with the patients’ prognosis at week 4 (**Table 3**), the GBS-DS at admission (OR = 0.22, *p* = 0.035) and MRC sum scores at peak (OR = 1.22, *p* = 0.035) predicted unfavorable outcomes at 12 weeks (**Table 4**). However, multivariate regression analysis did not find any association between these factors, as well as age, gender, antecedent illness, treatment interventions, and prognosis.

**Table 3:** Factors associated with GBS-DS ≤ 2 at week 4.

Factors	Univariate analysis		Multivariate analysis	
	OR (95%CI)	*P*	OR (95%CI)	*P*
Treatment				
Efgartigimod	1			
IVIG	0.00 (0.00 ~ Inf)	0.995		
Female	0.39 (0.07 ~ 2.30)	0.297		
Diarrhea	2.10 (0.21 ~ 20.64)	0.525		
Respiratory tract infection	0.51 (0.10 ~ 2.61)	0.423		
Age	1.00 (0.95 ~ 1.06)	0.992		
Days from onset to drug administration	0.84 (0.65 ~ 1.08)	0.171		
GBS-DS at admission	0.26 (0.07 ~ 1.02)	0.053		
GBS-DS at peak	0.00 (0.00 ~ Inf)	0.996		
MRC sum scores at admission	1.13 (1.01 ~ 1.26)	**0.030**	1.00 (0.84 ~ 1.19)	0.988
MRC sum scores at peak	1.15 (1.03 ~ 1.28)	**0.016**	1.13 (0.97 ~ 1.31)	0.114

Results are presented as odds ratio (OR) and 95% confidence intervals (CI).

GBS-DS, GBS disability score; IVIG, intravenous immunoglobulin; MRC, Medical Research Council. Bold values indicate statistical significance.

**Table 4:** Factors associated with GBS-DS ≤ 1 at week 12.

Factors	Univariate analysis		Multivariate analysis	
	OR (95%CI)	*P*	OR (95%CI)	*P*
Treatment				
Efgartigimod	1			
IVIG	0.00 (0.00 ~ Inf)	0.995		
Female	0.51 (0.11 ~ 2.44)	0.397		
Diarrhea	3.00 (0.31 ~ 28.84)	0.341		
Respiratory tract infection	1.00 (0.23 ~ 4.37)	1.000		
Age	1.02 (0.97 ~ 1.08)	0.381		
Days from onset to drug administration	0.86 (0.68 ~ 1.08)	0.193		
GBS-DS at admission	0.39 (0.13 ~ 1.21)	0.103		
GBS-DS at peak	0.22 (0.06 ~ 0.90)	**0.035**	0.40 (0.08 ~ 1.94)	0.253
MRC sum scores at admission	1.08 (0.99 ~ 1.17)	0.072		
MRC sum scores at peak	1.09 (1.01 ~ 1.18)	**0.035**	1.06 (0.95 ~ 1.17)	0.279

Results are presented as odds ratio (OR) and 95% confidence intervals (CI).

GBS-DS, GBS disability score; IVIG, intravenous immunoglobulin; MRC, Medical Research Council. Bold values indicate statistical significance.

## 4 Discussion

Our results revealed that in GBS patients with disability, efgartigimod showed numerically better short-term (week 4) and long-term (week 12) clinical outcomes than IVIG. This suggests that efgartigimod may represent a promising potential therapeutic option for GBS patients with disability, although these findings require further validation in larger studies. Furthermore, efgartigimod was well tolerated in our 12 patients; however, two patients experienced headaches, and no significant reduction in albumin levels was observed after treatment.

The GBS-DS has good clinical utility, and its classification criteria are highly consistent with the actual functional status of patients. Additionally, week 4 is a critical time point for patients with GBS to transition from acute progression to subacute plateau; evaluation at this time point can effectively evaluate the clinical effect of early treatment intervention. Therefore, we selected the GBS-DS score at week4 as the primary outcome, consistent with previous randomized controlled trials (4). In addition, given that some patients had a relatively short disease duration at enrollment, long-term prognostic assessment was not feasible. Accordingly, week 12 was designated as an intermediate follow-up time point to preliminarily evaluate the sustainability of treatment effects. Hence, future prospective studies with larger sample sizes and extended follow-up periods are warranted to further validate the long-term efficacy and safety of efgartigimod in patients with GBS.

Moreover, 70% of patients have a history of infection, and GBS represents a model of post-infectious autoimmune disease (14). Primary infection causes the anti-ganglioside antibody production, represented by GM1 and GD1a in acute motor axonal neuropathy (AMAN) and GQ1b in Miller Fisher syndrome (MFS). Furthermore, they cause axonal damage in the ganglia and nerve endings, leading to reversible conduction blockage, which is clinically well restored. In subsequent axonal degeneration, clinical recovery remains poor (15, 16). GBS encompasses two main subtypes: acute inflammatory demyelinating polyneuropathy (AIDP), which primarily affects the myelin sheaths, and AMAN, which primarily affects the axons. Variable pathophysiology in AMAN and AIDP leads to different clinical and neurophysiological recovery patterns. Recovery in AIDP depends on the degree of myelin regeneration and secondary axonal degeneration, whereas in AMAN, recovery relies on the extent of axonal changes caused by antibody deposition (1). Therefore, it is crucial to use immunotherapy to remove antibodies in the early stages of GBS. However, to date, treatment for GBS is still guided by trial results from over 30 years ago. Both IVIG and PE are effective in improving the prognosis of GBS patients by accelerating their recovery. However, neither IVIG nor PE can prevent the progression of GBS or reduce the extent of nerve damage (17). Furthermore, these two therapies face challenges, such as high costs, limited availability, and side effects. New strategies are also needed for patients who do not respond to PE or IVIG treatments. Various animal studies have reported that FcRn inhibition can reduce IgG levels. The administration of FcRn inhibitors significantly reduces antibody levels in mice, suggesting that FcRn inhibitors can treat GBS cases (7). Our research also confirms this point.

In this research, the number of days required to improve one functional grade was similar for efgartigimod and IVIG (10.0 days vs. 13.5 days), indicating that efgartigimod is equally effective as IVIG in preventing the disease progression. Notably, 100% of patients treated with efgartigimod were able to walk unassisted and run at weeks 4 and 12, respectively. This indicates that efgartigimod is beneficial in shortening the course of the disease and improving prognosis in GBS patients with disability. However, regression analysis did not reveal any differences between IVIG and efgartigimod in improving prognosis, likely due to our limited sample size. Since the efgartigimod group included three MFS-GBS overlap syndrome patients, they displayed superior MRC scores than the IVIG group at admission.

MFS is a variant of GBS, characterized by ophthalmoplegia, ataxia, and areflexia. Although simple MFS is not life-threatening, severe dizziness can cause patients to be bedridden, significantly affecting their quality of life. In some cases, MFS may coexist with other features such as limb weakness, paresthesia, and facial paralysis, which is considered MFS-GBS overlap syndrome and leads to a prolonged course of disease (18). A study on 50 patients showed that PE was ineffective in the amelioration of ataxia and ophthalmoplegia (19). In another study involving 92 patients, IVIG slightly accelerated the time between the onset of ophthalmoplegia and ataxia, as well as the time of symptom improvement, but did not improve patient prognosis (20). However, efgartigimod has demonstrated efficacy in alleviating ophthalmoplegia. In a relevant study, two MFS-GBS overlap syndrome patients who had failed to respond adequately to IVIG experienced rapid improvement in ophthalmoplegia (on days 2 and 4 post-efgartigimod), along with improved GBS disability scores, and the symptoms disappeared on the 23rd and 30th days of the disease course (8). In our study, there were three MFS-GBS overlap syndrome patients in the efgartigimod group; among the two cases, the ophthalmoplegia began to improve on the 5th and 8th day after efgartigimod administration. All three patients displayed normal eye movement within one month of onset. Notably, one of them reported the absence of symptoms approximately 10 days after onset, which was significantly shorter than the three months previously reported for the alleviation of ophthalmoplegia (20, 21). However, given the small sample size of MFS-GBS overlap syndrome patients in this study, these findings need to be interpreted with caution.

Respiratory dysfunction is a life-threatening clinical manifestation that occurs in 6% to 33% of GBS patients and leads to a poor prognosis (22). Intubation leads to various complications like pneumonia, pulmonary embolism, tracheal stenosis, etc. (23) In this study, the breathlessness of two respiratory insufficiency patients improved on the third day post-efgartigimod administration, avoiding the risk of subsequent intubation and ICU admission. In previous studies, GBS patients on IVIG had a significantly lower possibility of requiring ventilation and a shorter ventilation duration (15.2 days vs. 22.6 days) compared to those treated with PE (12). However, it is regrettable that due to our limited sample size, and considering that patients requiring mechanical ventilation and PE in our center need to be admitted to the ICU and typically require sedation, which will seriously interfere with the clinical assessment, so patients receiving mechanical ventilation and PE are ultimately excluded from this study. This limitation prevented a direct comparison of the efficacy differences among efgartigimod, IVIG, and PE in improving respiratory function. Therefore, future prospective studies with larger sample sizes are needed to systematically evaluate the therapeutic efficacy and safety of these three treatment modalities in GBS patients with concomitant respiratory dysfunction.

Clinical research has demonstrated that repeated administrations of efgartigimod at a dose of 10 mg/kg induce the maximal reduction in serum IgG levels. Elevated dosages fail to bring about a significant further decrease in IgG concentrations while concurrently elevating the risk of adverse events (24). Given the characteristics of GBS progression, most of our patients received two courses of efgartigimod (10mg/kg), with 3-7-day gaps between the doses. Another case report revealed a treatment regimen of administering AMAN once a week for four consecutive weeks (9), as well as sequential efgartigimod administration following inadequate response to IVIG (8). In addition, the optimal treatment of GBS with efgartigimod should be studied in the future.

Moreover, advanced age, diarrhea, and high MRC scores at admission are associated with poor outcomes (25), however, we did not observe any such association. This phenomenon may be related to the small sample size of our study, which limits the statistical power of multivariate analysis to find true associations. With regard to gender, statistical analysis found that the incidence rate in males was higher than that in females, with a ratio of 1.5:1 (26), However, in our study, the proportion of female patients was higher (59%), especially in the IVIG group, with a significantly higher proportion of women than in the efgartigimod group (77% vs. 25%, *P* = 0.005). Although studies have suggested that gender may differ in the clinical presentation and recovery pattern of GBS, the available retrospective evidence does not identify gender as an independent poor prognostic factor. Therefore, although there is an imbalance in the gender distribution between the two groups, its confounding effect on the primary outcome of this study may be limited (27, 28).

These limitations (including small sample size, single-center design, and baseline imbalance between groups) may partly explain why we failed to reproduce some of the prognostic associations reported in the literature, and suggest that the conclusions of this study should be interpreted with caution. Furthermore, we are susceptible to recall bias and confounding factors due to our retrospective design. The relatively short follow-up period also made assessment of long-term efficacy and safety difficult. Future larger-scale, multicenter, prospective studies are needed to further verify the efficacy of efgartigimod in GBS and to further explore the role of factors such as gender in prognosis.

## 5 Conclusion

This preliminary retrospective study implies that efgartigimod has potential efficacy and safety for GBS with disability, and shows tentative benefits in improving ophthalmoplegia and respiratory insufficiency symptoms. Future well-designed prospective trials with larger sample sizes and multicenter participation are urgently needed to validate our preliminary results, explore the optimal treatment regimen for GBS therapy with efgartigimod, and clarify its efficacy in specific GBS subtypes, severe manifestations such as respiratory insufficiency, as well as its potential effects on electrophysiological indices of GBS patients.

## Data Availability

The original contributions presented in the study are included in the article/supplementary material. Further inquiries can be directed to the corresponding authors.
